# Plasma PTX3, MCP1 and Ang2 are early biomarkers to evaluate the severity of sepsis and septic shock

**DOI:** 10.1111/sji.12823

**Published:** 2019-10-07

**Authors:** Rui Tian, Xiaoli Wang, Tingting Pan, Ranran Li, Jiahui Wang, Zhaojun Liu, Erzhen Chen, Enqiang Mao, Ruoming Tan, Yang Chen, Jialin Liu, Hongping Qu

**Affiliations:** ^1^ Department of Critical Care Medicine Ruijin Hospital Shanghai Jiao Tong University School of Medicine Shanghai China; ^2^ Department of Emergency Ruijin Hospital Shanghai Jiao Tong University School of Medicine Shanghai China

**Keywords:** Ang2, MCP1, pro‐inflammatory cytokine, PTX3, sepsis

## Abstract

Sepsis is associated with significant mortality. Early diagnosis and prognosis of patients with sepsis is still a difficult clinical challenge. In this study, the ability of plasma PTX3 (pentraxin 3), MCP1 (monocyte chemoattractant protein 1) and Ang (angiopoietin)1/2 was investigated to evaluate the severity of sepsis. Blood samples were obtained from 43 patients with sepsis. A total of 33 post‐surgery patients with infections and 25 healthy individuals served as controls. The results showed that plasma PTX3, MCP1 and Ang2 significantly increased in patients on the first day of septic shock onset, while sepsis patients had significantly higher Ang2 level, compared with controls. Furthermore, PTX3, MCP1 and Ang2 had high AUROC values in patients with septic shock on the first day of sepsis onset. The findings suggest that PTX3, MCP1 and Ang2 maybe early predictors to evaluate the severity of sepsis and septic shock with the latest Sepsis 3.0 definitions.

## 1 INTRODUCTION

Sepsis is a serious disorder of the body against bacterial or other pathogens and ultimately induces multiple organ dysfunction. It is one of the leading causes of death in patients in intensive care unit (ICU).^1^, ^2^ In the early stage of sepsis, innate immunity is activated and subsequently ignites a cascade of immune responses within the cells.^3^ The disorders of immunity function would lead to an autoamplifying cytokine production and eventually lead to organ dysfunction.^3^, ^4^ Since the introduction of Sepsis 3.0 definitions focuses on organ damage, traditional biomarkers such as CRP (C‐reactive protein) and PCT (procalcitonin) are inconsistent in terms of diagnostic capabilities.^5^, ^6^, ^7^ Therefore, better biomarkers for early diagnosis of sepsis are urgently needed.

Among all soluble pattern recognition molecules, pentraxin family plays an important role in the regulation of inflammation. CRP, commonly used clinically to help detect inflammatory responses, is also a member of pentraxin family. Cytokines such as tumour necrosis factor‐⍺ (TNF‐⍺) and interleukin‐1 (IL‐1) can effectively induce the release of PTX3 by several types of cells such as vascular endothelial cells, monocytes and neutrophils.^8^, ^9^, ^10^ Recent studies showed that PTX3 had a non‐redundant role in the regulation of inflammation associated with cancer and various infections such as *Klebsiella* pneumoniae and *E coli*..^11^, ^12^

Blood monocytes serve as the first line of defence against pathogen invasion and are equipped to respond to infection.^13^ Monocyte chemoattractant protein 1 (MCP1), also known as C‐C motif chemokine ligand 2 (CCL2), is an initiating cytokine of the inflammatory cascade secreted by endothelial cells, monocytes and other cells.^14^ MCP1 affects monocyte migration together with subsequent macrophage polarization and recruitment of immune cells to the site of injury.^15^, ^16^ An increasing number of evidence have shown that in animal models of sepsis, the high levels of MCP1 are strongly correlated with organ dysfunction and mortality.^17^

An important characteristic of sepsis is endothelial activation. The endothelial‐specific angiopoietin (Ang)‐tyrosine kinase (Tie) system has been considered as an important mechanism in regulating endothelial activation of sepsis and as a possible determinant of injury severity.^18^ Angiopoietin 1 (Ang1) is released by pericytes, and it enhances the stability of formed vessels by binding to its receptor Tie2 in endothelial cells.^19^ Ang2 is stored in Weibel‐Palade bodies in endothelial cells. As the antagonist of Ang1, Ang2, bound to Tie2, promotes the loss of barrier integrity, resulting in vascular leak and organ dysfunction in sepsis.^20^

Till now, the study of clinical significance of biomarkers in the setting of Sepsis 3.0 definitions is limited. The aim of this study was to investigate the diagnostic value of PTX3, MCP1 and Ang1/Ang2 in patients with sepsis and septic shock on the first day of sepsis onset according to the latest Sepsis 3.0 definitions.

## 2 MATERIALS AND METHODS

### 2.1 Selection of patients and controls

Forty‐three patients diagnosed with sepsis and septic shock from May 2017 to September 2018 were recruited from the intensive care unit (ICU) of Ruijin Hospital. Twenty‐five healthy volunteers were included as controls. All of the control participants matched with the patient population in terms of age and sex. Thirty‐three post‐surgery patients who were transferred to the ICU after surgery with infections were enrolled and served as another control group.

### 2.2 Data collection

The criteria for sepsis and septic shock were in accordance with the Sepsis 3.0 definitions: Patients were assigned to the sepsis group if they had life‐threatening organ dysfunction which was represented by an increase in the Sequential Organ Failure Assessment (SOFA) score of two points or more after infection. When patients need vasopressor requirement to maintain a mean arterial pressure of 65 mm Hg or greater, and the serum lactate level was <2 mmol/L in the absence of hypovolaemia, they were classified as septic shock.^21^

According to the new guidelines, lactate levels were assessed for all patient groups. Disease severity in the ICU was documented according to the Acute Physiology and Chronic Health Evaluation II (APACHE II) and the Sequential Organ Failure Assessment (SOFA) score.

All patients’ data, such as creatinine levels, haemoglobin, haematocrit, white blood cell count, platelet count, CRP, bilirubin, sodium, potassium, urea, PCT, body temperature, respiratory rate, heart rate and blood pressure, were documented from the patients’ files. Coagulation and fibrinolysis indices such as activated partial thromboplastin time (APTT), prothrombin time (PT), thrombin time (TT), international normalized ratio (INR), fibrinogen (Fg) and D‐dimer were also documented from the patients’ files. Additionally, prior medical history, age, sex, body weight and the germ spectrum were documented.

### 2.3 Ethics statement

The study was conducted in accordance with the Declaration of Helsinki, and the protocol was approved by the Ruijin Hospital Ethics Committee, Shanghai Jiaotong University School of Medicine, China (Reference Number: 201349 released on 05 July 2013). Written informed consent was obtained from all participants or their relatives.

### 2.4 Blood samples

Blood samples for measurements were taken within 24 hours after clinical onset of sepsis or after surgery (day 1). Within 30 minutes, all blood samples were centrifuged at 2500 × *g* at 4°C for 10 minutes. Plasma was separated and aliquoted. The aliquoted samples were stored at −80°C until analysis.

### 2.5 Biomarker measurements

Circulating levels of inflammatory and immunity markers (PTX3, MCP1, Ang1, Ang2, IL‐6, IL‐8, IL‐10 and TNF‐⍺) were measured in plasma samples by using Luminex techniques in parallel (LXSAHM‐08, R&D) according to the manufacturer's instructions.

### 2.6 Statistical analysis

All statistical analyses were performed by using IBM SPSS Statistics (version 23.0). Figures were prepared by using GraphPad Prism version 6.0 (GraphPad Software). Continuous variables of the data were presented as mean ± standard error of mean (SEM) or median with interquartile range (IQR), and categorical data were presented with frequencies and percentage. All variables were not strictly normal distribution. Thus, we applied Spearman's rank correlation with non‐parametric data to test the association between biomarkers blood levels and traditional immune parameters. The receiver operating characteristic (ROC) curve was used to assess accuracy. All multiple groups were calculated by Kruskal‐Wallis test. All analyses were exploratory, and a two‐tailed *P*‐value of < .05 was taken as a cut‐off for statistical significance.

## 3 RESULTS

### 3.1 Study of population characteristics in sepsis, septic shock and post‐surgery patients

A total of 76 patients and 25 healthy controls have been enrolled into the study, in which 17 patients suffered from sepsis, 26 patients had septic shock and 33 were post‐surgery patients. The mean value for the APACHE II in septic shock and sepsis patients was 21 and 15, respectively, indicating relatively high severity. SOFA score in septic shock and sepsis patients was 10 and 5, respectively. The most common sites of infection in septic shock patients were abdomen (approximately 54%) and blood (approximately 27%). In sepsis patients, the most common sites of infection were also abdomen (approximately 65%) and blood (approximately 47%). Laboratory results including WBC, CRP, PCT and ICU parameters were also shown in Table 1.

**Table 1:** Tian et al Characteristics of sepsis and post‐surgery patients

Characteristics	Sepsis	Septic Shock	Post‐Surgery	Healthy Controls
Age, mean (range)	54 (29‐90)	63 (19‐86)	68 (22‐89)	38 (26‐54)
Gender (female/male)	6/11	7/19	12/21	12/13
Site of infection, n (%)				
Lung	2 (12)	5 (19)	0 (0)	–
Abdominal	11 (65)	14 (54)	1 (3)	–
Blood	8 (47)	7 (27)	1 (3)	–
Others	4 (24)	6 (23)	1 (3)	–
Laboratory values, mean ± SEM				
WBC (10^9^/L)	13.2 ± 1.8	21.2 ± 2.8	10.1 ± 0.9	–
Platelets (10^9^/L)	187 ± 25.5	145 ± 16.7	174 ± 14.6	–
Lactate, (mmol/L)	2.8 ± 0.5	3.5 ± 0.7	1.7 ± 0.3	–
CRP, (mg/L)	136 ± 28.5	161.2 ± 17.5	39.4 ± 11.8	–
Procalcitonin, (ng/mL)	26.3 ± 13.2	78.5 ± 19.1	0.9 ± 0.5	–
Creatinine, (umol/L)	162 ± 51.5	170 ± 24.4	75 ± 5.2	–
Urea, (mmol/L)	10.5 ± 1.7	14.4 ± 1.7	10.1 ± 4.6	–
ICU parameters, mean ± SEM				
ICU days	39 ± 9.6	29 ± 7.5	7 ± 2.7	–
Ventilation days	4 ± 1.7	14 ± 6.6	3 ± 1.2	–
APACHE II	15 ± 1.3	21 ± 1.7	11 ± 0.9	
SOFA score	5 ± 0.6	10 ± 0.8	0.67 ± 0.2	–
Respiratory failure, n (%)	5 (29)	13 (48)	1 (3)	–
AKI, n (%)	4 (24)	13 (48)	0 (0)	–
28‐day mortality, n (%)	0 (0)	4 (15)	1 (3)	–

Note

Data are presented as n (percentages), mean ± SEM.

Abbreviations: AKI, acute kidney injury; APACHE II, Acute Physiology and Chronic Health Evaluation II; CRP, C‐reactive protein; n, numbers; SEM, mean ± standard error of mean; SOFA, Sequential Organ Failure Assessment; WBC, white blood cell count.

### 3.2 Distribution of plasma MCP1, PTX3, Ang1 and Ang2

The biomarker plasma levels in septic shock patients on the first day were the highest in these four groups as shown in Table 2. The median value of plasma MCP1, PTX3, Ang1 and Ang2 in septic shock patients was 661.32, 14 635.50, 10 400.00 and 8213.00 pg/mL, respectively, while the median value of MCP1, PTX3 and Ang1 in sepsis group was similar to the post‐surgery group. Moreover, the expression of plasma Ang2 levels in septic shock patients (8213.00 pg/mL) was approximately two times higher than that in sepsis group (5824.00 pg/mL).

**Table 2:** Tian et al Data analysis of MCP1, PTX3, Ang1 and Ang2 in patients with sepsis, septic shock, post‐surgery and healthy controls

Variable Median (IQR) (pg/ml)	Sepsis	Septic shock	Post‐Surgery	Healthy Controls
MCP1	310.10 (229.64,586.61)	661.32 (299.66,1423.00)	348.93 (254.58,645.25)	157.06 (122.73,184.63)
PTX3	5430.00 (1431.00,10 114.50)	14 635.50 (7364.50,30 485.00)	1939.00 (1159.00,4311.00)	83.82 (17.22,226.53)
Ang1	11 868.00 (3198.00,29 334.50)	10 400.00 (4928.00,23 093.50)	13 383.00 (6134.00,17 420.00)	2759.00 (1897.50,4781.00)
Ang2	5824.00 (3402.50,8947.00)	8213.00 (4681.50,10 133.25)	2190.00 (1707.50,3433.50)	2883.00 (844.53,1692.50)

Note

Data are presented as median (IQR).

Abbreviation: IQR, interquartile range.

**Figure 1:**
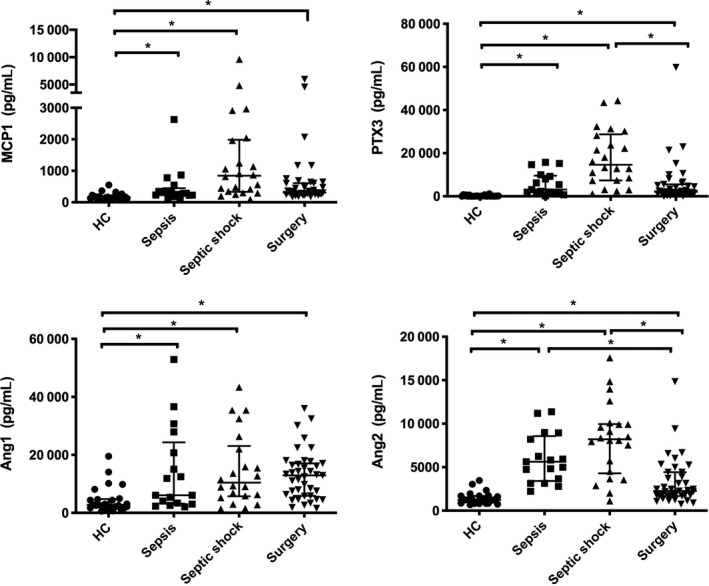
Tian et al Comparative analysis of MCP1, PTX3, Ang1 and Ang2 in patients with sepsis, septic shock, post‐surgery and healthy controls. Concentrations of MCP1, PTX3, Ang1 and Ang2 in plasma from HC (healthy controls, filled circles), sepsis patients (filled squares), septic shock patients (filled triangles) and post‐surgery patients (inversed filled triangles) were measured by Luminex. Data are presented as median (IQR). *P < .05

Figure 1 showed the distribution of MCP1, PTX3, Ang1 and Ang2 in sepsis, septic shock and post‐surgery patients as well as in healthy control. The levels of MCP1 and Ang1 in all other groups were higher than the healthy controls (*P* < .05). The PTX3 levels in septic shock patients were significantly higher, compared with post‐surgery and healthy control groups (*P* < .05). Plasma Ang2 levels of sepsis patients and septic shock patients were considerably higher than those in healthy control and post‐surgery group (*P* < .05). The data indicate that plasma PTX3, MCP1 and Ang2 have higher levels among sepsis patients, especially in septic shock patients

### 3.3 Correlations between traditional immune parameters and MCP1, PTX3, Ang1 and Ang2

Correlations between MCP1, PTX3, Ang1, Ang2 and traditional immune parameters including pro‐inflammatory and anti‐inflammatory cytokines in patients were analysed. The correlations in all sepsis patients could be found in Table 3. MCP1 had significant correlation with TNF‐⍺ (*r* = .576, *P* < .05), IL‐6 (*r* = .680, *P* < .05), IL‐8 (*r* = .706, *P* < .05) and IL‐10 (*r* = .526, *P* < .05). PTX3 had strong correlations with TNF‐⍺ (*r* = .388, *P* < .05), IL‐6 (*r* = .525, *P* < .05), IL‐8 (*r* = .529, *P* < .05) and IL‐10 (*r* = .255, *P* < .05). Plasma Ang2 had statistically significant correlations with TNF‐⍺ (*r* = .408, *P* < .05) and IL‐6 (*r* = .316, *P* < .05).

**Table 3:** Tian et al Correlations of MCP1, PTX3, Ang1 and Ang2 with traditional immune parameters in sepsis and septic shock patients

Variable	Correlation (*r*)	*P*
Ang1		
TNF‐α	−.031	.84
IL‐6	.060	.70
IL‐8	.054	.73
IL‐10	.072	.65
Ang2		
TNF‐α	.408	<.05*
IL‐6	.316	<.05*
IL‐8	.289	.06
IL‐10	.275	.08
MCP1		
TNF‐α	.576	<.05*
IL‐6	.680	<.05*
IL‐8	.706	<.05*
IL‐10	.526	<.05*
PTX3		
TNF‐α	.388	<.05*
IL‐6	.525	<.05*
IL‐8	.529	<.05*
IL‐10	.255	<.05*

* *P *< .05.

### 3.4 The levels of Ang and coagulation indices between septic and post‐surgery patients

As Ang2 promoted the loss of barrier integrity, which can result in vascular leak in sepsis, we analysed the distribution of coagulation indices in different groups. As shown in Table 4, there were significant differences among the three groups regarding the coagulation and fibrinolysis indices, including APTT, PT, INR, Fg and D‐dimer (*P < *.05 respectively). Besides, the level of platelets in septic shock patients was significantly lower than that in sepsis and post‐surgery patients (*P* = .053).

**Table 4:** Performance of variables in predicting the severity of septic shock

Variable	AUC ROC	95% CI	*P*‐value	Sensitivity (%)	Specificity (%)	Cut‐off (pg/mL)
MCP1	0.716	0.564‐0.868	.0059	69.23	70.59	382
PTX3	0.798	0.666‐0.921	<.0001	50.00	100.00	15 877
Ang1	0.501	0.320‐0.683	.9904	69.23	47.06	6182
Ang2	0.631	0.464‐0.799	.1288	42.31	88.24	9047

Furthermore, we analysed the correlations between Ang and coagulation indices. As shown in Table 5, Ang2 had significant correlation with APTT (*r* = .439, *P* < .05), PT (*r* = .451, *P* < .05), INR (*r* = .447, *P* < .05), Fg (*r* = .263, *P* < .05) and D‐dimer (*r* = .366, *P* < .05).

**Table 5:** Tian et al Performance of variables in predicting septic shock

Variable	AUC ROC	95% CI	*P*‐value	Sensitivity (%)	Specificity (%)	Cut‐off (pg/mL)
IL‐6	0.713	0.551‐0.874	.0110	80.77	58.82	26
Ang2	0.631	0.464‐0.799	.1288	42.31	88.24	9047
Combine	0.687	0.522‐0.852	.0287	73.08	64.71	–
MCP1	0.716	0.564‐0.868	<.0001	69.23	70.59	382
Combine	0.721	0.570‐0.871	.0045	46.15	94.12	–
PTX3	0.798	0.666‐0.921	<.0001	50.00	100.00	15 877
Combine	0.798	0.666‐0.929	<.0001	53.85	100.00	–

### 3.5 Selected biomarkers discriminated sepsis and septic shock according to the latest Sepsis 3.0 definitions

**Figure 2:**
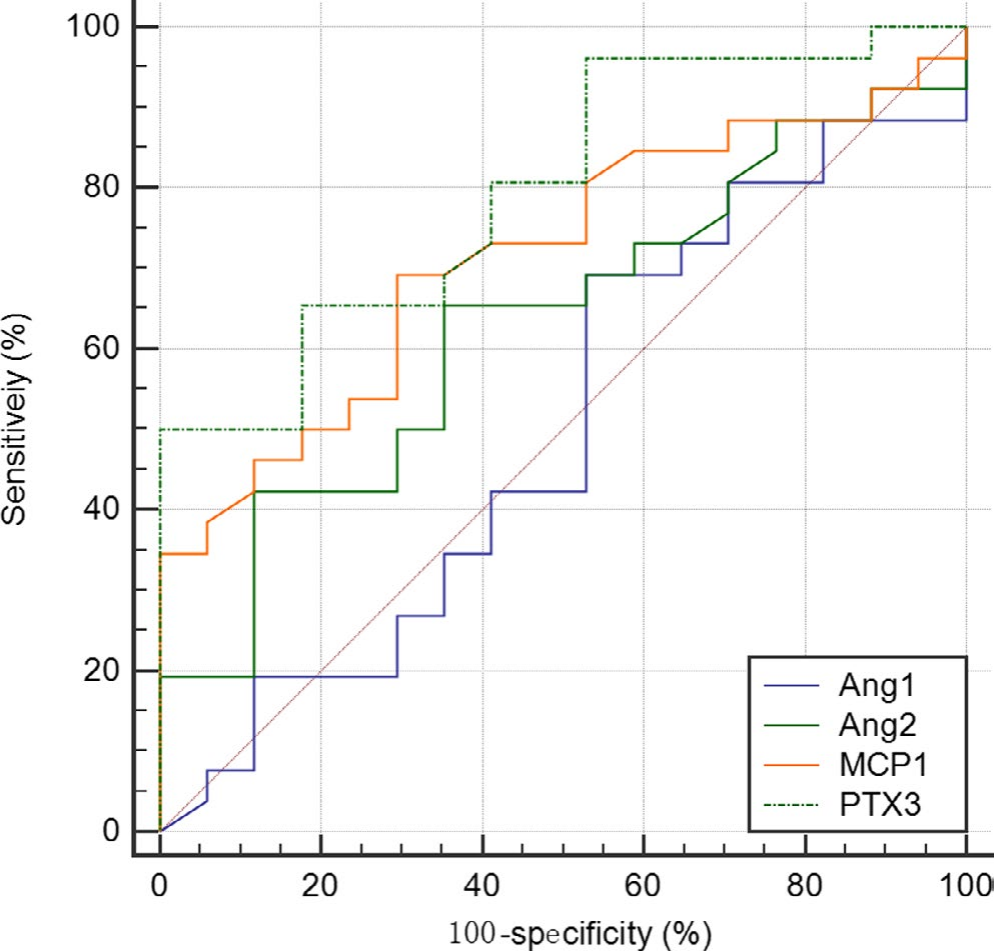
Tian et al Receiver operating characteristic (ROC) curves for predicting septic shock by plasma level of MCP1, PTX3, Ang1 and Ang2

Whether these four biomarkers could be used to differentiate sepsis and septic shock was further investigated. It was found that the AUROC values were 0.716 (95% CI 0.564‐0.868, *P* = .0059) for MCP1 expression, 0.798 (95% CI 0.666‐0.921, *P* < .0001) for PTX3 expression, 0.501 (95% CI 0.320‐0.683, *P* = .9904) for Ang1 expression and 0.631 (95% CI 0.464‐0.799, *P* = .1288) for Ang2 expression in septic shock patients (Figure 2, Table 6). Moreover, as IL‐6 was one of the most common clinical cytokines for predicting infections, the AUROC values of IL‐6 in septic shock patients were 0.713 (95% CI 0.551‐0.874, Table 7). The biomarkers combined with IL‐6 did not have a higher AUROC value than the individual biomarkers.

**Table 6:** Tian et al Data analysis of APTT, PT, TT, INR, Fg and D‐dimer in patients with sepsis, septic shock and post‐surgery

Variable Median (IQR)	Sepsis	Septic shock	Post‐surgery	*P* value
PLT (10^12^/L)	186.00 (121.50,294.00)	118.50 (83.00,116.75)	160.00 (109.00,238.00)	.053
APTT (s)	31.80 (28.35,37.20)	39.10 (32.00,48.36)	28.00 (25.70,31.20)	<.05
PT (s)	12.90 (11.85,14.00)	16.85 (14.37,18.475)	13.00 (12.20,14.05)	<.05
TT (s)	16.90 (16.30,17.85)	17.25 (16.10,18.95)	17.30 (16.40,18.00)	.894
INR	1.06 (1.00,1.19)	1.45 (1,23,1.58)	1.10 (1.03,1.19)	<.05
Fg (g/L)	4.40 (2.95,5.05)	4.05 (2.60,4.80)	2.90 (2.30,3.45)	<.05
D‐dimer (mg/L)	5.50 (2.99,9.41)	7.05 (4.25,14.65)	3.11 (1.72,4.90)	<.05

Note

Data are presented as median (IQR).

**Table 7:** Tian et al Correlations of Ang1 and Ang2 with coagulation index in sepsis and septic shock patients

Variable	Correlation (*r*)	*P*
Ang1		
APTT	−.092	.432
PT	−.031	.790
TT	.153	.187
INR	−.018	.876
Fg	−.028	.810
D‐dimer	−.049	.677
Ang2		
APTT	.439	<.05*
PT	.451	<.05*
TT	−.032	.787
INR	.447	<.05*
Fg	.263	<.05*
D‐dimer	.366	<.05*

* *P *< .05.

## 4 DISCUSSION

The present study evaluated the diagnostic values of MCP1, PTX3, Ang1 and Ang2 in patients with sepsis and septic shock according to the latest Sepsis 3.0 definitions. The results showed that MCP1, PTX3 and Ang2 had higher levels in sepsis patients, especially in those with septic shock, compared with controls. Moreover, PTX3, MCP1 and Ang2 had high AUROC values in patients with septic shock on the first day of sepsis onset.

Innate immunity is the first line of host defence. Compared with CRP, PTX3, produced only by the liver, is secreted by different cell types corresponding to infection, pro‐inflammatory stimulation and endotoxemia.^22^, ^23^, ^24^ Dias et al showed that PTX3 transgenic mice were more resistant to sepsis.^25^ Our study revealed that plasma PTX3 levels could be used to evaluate sepsis and septic shock on the first day of sepsis onset. In addition, our study confirmed that circulating PTX3 levels were elevated in sepsis patients and were even higher in septic shock patients, the results of which were similar to previous studies of others.^26^ It was also found that PTX3 levels in sepsis and septic shock patients had much more significant correlations with traditional immune parameters than in post‐surgery patients. Pro‐inflammatory cytokines such as TNF‐⍺ and IL‐1β were excessively activated since sepsis was induced by severe infections, which induced the expression of PTX3.^27^ Aaton et al showed that PTX3 regulated the development of atherosclerosis by enhancing the production of IL‐10 in vitro. However, the PTX3 stimulation did not result in the production of pro‐inflammatory cytokines IL‐6 and TNF‐⍺.^28^ The study presented significant and valuable AUCs for discriminating sepsis or septic shock from healthy and post‐surgery controls. Previous studies showed that PTX3 had significant correlations with APACHE II score and SOFA score in sepsis and septic shock patients.^26^, ^29^ Moreover, the plasma PTX3 level could predict 90 mortalities in sepsis patients.^29^ Thus, novel biomarker such as PTX3 might help improve the early diagnosis of sepsis and septic shock.

MCP1 plasma levels in septic shock patients were much higher than those in other groups. This study demonstrated that plasma MCP1 levels presented significant and valuable AUCs for discriminating septic shock from healthy and post‐surgery controls. Several studies have reported that MCP1 also had the ability to regulate inflammatory progression and the production of pro‐inflammatory cytokines in adipocytes, ankylosing spondylitis and cancer.^30^, ^31^, ^32^ Our results showed a positive correlation between plasma MCP1 and cytokines (TNF‐α, IL‐6, IL‐8 and IL‐10) in sepsis. Moreover, Zhu T et al found that plasma MCP1 maybe a useful biomarker in predicting the prognosis of sepsis.^33^ In the study by Barre M et al, the levels of MCP1 showed prognostic value for short‐ and mid‐term mortality in patients with sepsis according to the Sepsis 3.0 definitions.^34^ Thus, the plasma levels of MCP1 could be an early marker in distinguishing septic shock, and high MCP1 levels maybe associated with poor prognosis in critically ill patients.^35^

Vascular endothelial cells lining in the inner side of vascular system play a significant role in maintaining vascular homeostasis. In the early stage of sepsis, excessive release of pro‐inflammatory cytokines and the adhesion of leucocytes disrupt endothelial cell integrity and eventually lead to vascular leakage, disturbance of blood flow and sepsis‐induced organ failure.^18^ In severe infections and sepsis, persistent vascular damage and leakage can contribute to tissue hypoperfusion.

The angiopoietins are a family of secreted factors comprising Ang1, Ang2 and Ang3 (Ang4 in humans).^36^ Ang1 is produced and excreted into the blood by pericytes and smooth muscle cells, and it is also stored in platelets.^37^ Ang1 protects against vascular leakage, whereas Ang2 promotes increased vascular permeability. Recent reports associated Ang2 with disseminated intravascular coagulation in inflammatory diseases.^38^ In our research, Ang was compared with coagulation/fibrinolysis indices. The current findings showed that the coagulation indices (APTT, PT, INR and D‐dimer) in patients with sepsis and septic shock were significantly higher than those in patients with post‐surgery. However, the level of fibrinogen was lower in the sepsis group than in the septic shock group. All the results might demonstrate that Ang2 was involved in the progression of coagulation. Furthermore, it was found in our research that Ang1 had low ROC values, sensitivity and specificity in our recruited sepsis patients. Evidence in the literature suggests that the vessel‐destabilizing functions of Ang2 are main contributors to the septic phenotype.^39^, ^40^ Our results indicated that Ang2 was highly upregulated in the plasma of sepsis patients, and its expression strongly correlated with the severity of the disease. In the ROC analysis of sepsis patients, the ROC area, sensitivity and specificity of Ang2 were the best among the four biomarkers that were tested. David S et al found that Ang2 may contribute to the adverse outcomes in sepsis.^41^ Thus, it is concluded that high level of Ang2 (not Ang1) contributes to distinguish sepsis and septic shock, making Ang2 a worthwhile therapeutic target in sepsis.

There are several limitations in this study. First, the present study was performed as a single‐centre study. Second, we studied these markers in patients admitted to the ICU and most of them came from surgical department. Therefore, our results may not be applicable to patients with less severe illness. The group of patients included in this study was small, and future studies with larger cohorts are needed to validate these results.

Collectively, our data revealed that PTX3, MCP1 and Ang2 were highly upregulated in the plasma of sepsis, and the expression strongly correlated with the severity of sepsis. Besides, the level of Ang2 might be involved in the progression of coagulation during sepsis. Furthermore, PTX3, MCP1 and Ang2 maybe early predictors to evaluate the severity of sepsis and septic shock according to the latest Sepsis 3.0 definitions.

## CONFLICTS OF INTEREST

The authors declare no conflict of interest.

## AUTHOR CONTRIBUTIONS

Rui Tian and Xiaoli Wang: contributed equally to this work. Hongping Qu, Enqiang Mao, Eerzhen Chen, Rui Tian, Xiaoli Wang and Ranran Li: conceived and designed the experiments. Jiahui Wang, Ruoming Tan and Zhaojun Liu: performed the experiments. Jialin Liu: analysed the data. Tingting Pan, Jiahui Wang and Ruoming Tan: contributed reagents/materials/analysis tools. Hongping Qu and Ranran Li: supervised the study. Rui Tian and Xiaoli Wang: wrote the article. All authors approved the final draft submitted.
